# Effect of a 12-week exercise program on phase angle in women with breast cancer

**DOI:** 10.1007/s00520-025-09443-4

**Published:** 2025-04-21

**Authors:** Adrian Escriche-Escuder, José Manuel García-Almeida, Isabel María Vegas-Aguilar, Bella Pajares, Emilio Alba, Manuel Trinidad-Fernández, Cristina Roldán-Jiménez, Antonio Ignacio Cuesta-Vargas

**Affiliations:** 1 Department of Physiotherapy, University of Malaga, 3. PC: 29071C/Arquitecto Peñalosa, Málaga, Spain; 2 Instituto de Investigacion Biomedica, (IBIMA plataforma Bionand), Malaga, Spain; 3 Department of Endocrinology and Nutrition, Hospital Regional Universitario y Virgen de La Victoria, Málaga, Spain; 4 UGCI Oncológica Médica, Hospital Regional Universitario y Virgen de La Victoria, Málaga, Spain

**Keywords:** Bioelectrical impedance analysis, Functional capacity, Reactance, Resistance

## Abstract

**Purpose:**

The aims of this study were to analyze the effects of a 12-week exercise intervention on bioelectrical impedance analysis-derived phase angle (PhA), resistance (R), and reactance (Xc) in breast cancer survivors (BCS) and analyze the relationship between changes in bioelectrical impedance variables and changes in functional capacity and muscular strength.

**Methods:**

This was a prospective cohort study. Potentially eligible patients were recruited from the Medical Oncology Unit of the hospital. Female BCS older than 18 years were offered to participate in the study if they had previously undergone surgery for their primary tumor and there was no evidence of recurrence at the time of recruitment. A 12-week exercise program including resistance and endurance training was performed, including two weekly sessions led by a physical therapist. Measurements were performed at baseline and after 12 weeks, including PhA and function-related outcomes. The relationship between changes in PhA and function-related outcomes was assessed using the Pearson *r* correlation coefficient.

**Results:**

Sixty-seven BCS women were included in the analysis. A significant increase was found in PhA and functional outcomes after the intervention, as well as a significant decrease in R. Bivariate correlations showed a significant positive correlation between PhA and functional tests (Hand grip, *r* = 0.37 [*p* = 0.002], 30-Sit to Stand, *r* = 0.39 [0.002], respectively).

**Conclusion:**

A 12-week concurrent exercise program may be effective to improve PhA and R. Additionally, there appears to be a correlation between PhA and Xc with functional capacity outcomes. Finally, baseline PhA, Xc, and R values explained part of the 30-STS and hand grip tests variance at 12 weeks, which could suggest its importance in the prognosis.

**Supplementary Information:**

The online version contains supplementary material available at 10.1007/s00520-025-09443-4.

## Introduction

Breast cancer is the most commonly diagnosed cancer in the world, accounting for 11.6% of all new cancer cases, and remains the leading cause of cancer-related deaths among females [1]. Over the past three decades, medical advances have increased survival rates for this type of cancer by approximately 40% [2]. However, due to both the disease and the treatments used, breast cancer patients and survivors often experience an increase in body fat mass and a reduction in fat-free mass, particularly in skeletal muscle, which negatively affects their overall health [3, 4]. In this context, current research indicates that monitoring changes in body composition in this population can provide valuable insights for clinicians [5], as these alterations may be closely related to prognosis, diagnosis, and treatment outcomes [5].

Bioelectrical impedance analysis (BIA) has become one of the most widely used methods to study body composition, largely due to its non-invasive nature, affordability, portability, high reliability, and low inter-observer variability [6–8]. This evaluation technique works by passing a weak, alternating electrical current through the body, capitalizing on the fact that different tissues offer varying levels of impedance, which is defined as the resistance to the flow of electrical current [6, 9]. However, BIA is an indirect evaluation method that uses several regression equations to obtain various body composition parameters and that can generate errors in the estimation of the resulting values, being a limitation mainly in the case of those devices that do not offer raw parameters and use algorithms owned by manufacturers [10]. Conversely, the analysis of raw parameters, when these are provided by the device, allows for greater qualitative and quantitative analysis. Thus, the availability of BIA raw parameters allows analyzing these parameters themselves limiting the use of equations, calculating derived parameters (such as the BIA-derived phase angle [PhA]), or using specific formulas and equations for populations with special characteristics [10].

In this context, the use of the raw parameters’ resistance (R) and reactance (Xc) and the derived parameter PhA has gained popularity, also in patients with breast cancer [11–15]. The raw parameter R represents the opposition of tissue to the flow of electric current [16]. Differently, Xc represents cell membrane capacitance and can be interpreted as an indirect measure of intracellular volume [16]. Thus, PhA is calculated as the ratio between R and Xc, and it represents the angle between the impedance vector (formed by R and Xc) and the *X*-axis [9].

PhA has been shown to provide useful clinical information about the general health status of individuals and is considered an important prognostic indicator of survival and quality of life in cancer survivors [11, 17, 18]. It offers insights into cell membrane integrity, body fluid distribution, nutritional status, and functional capacity [9], and there seem to be clear differences between the values found in healthy and health-compromised individuals [16]. Higher PhA values are typically observed in healthy individuals and are suggestive of elevated Xc, which is associated with intact cell membranes, or lower R, related to higher body water and electrolyte levels [19]. In contrast, lower PhA values are indicative of low Xc, which is associated with compromised cell membrane integrity or cell death, or high R, which may signal malnutrition [20]. As a result, reduced PhA values are linked to poor health, reduced quality of life, and an unfavorable prognosis, particularly in patients with cancer [12, 15, 21]. It has been suggested that cancer and its treatments contribute to cell membrane damage [22], inflammation, and cancer-related cachexia [18], all of which result in a decrease in body cell mass [23]. These factors could directly translate into a decrease in PhA, which is consistent with current evidence showing significantly lower PhA values in cancer patients or survivors compared to their healthy, age-matched peers [18].

Previous literature has suggested that exercise and nutritional interventions may help reverse these adverse changes in cancer patients and survivors: On the one hand, it is hypothesized that nutritional approaches may reduce R by increasing electrolyte-containing water [24]; on the other hand, exercise may potentially produce adaptations that enhance cell membranes integrity, leading to an increase in Xc [18].

In this context, previous studies have analyzed the effects of exercise programs on cancer patients [9, 18]. In general, evidence suggests that resistance and/or endurance training can increase PhA in cancer survivors [9]. However, the literature remains limited and insufficient to discern the most effective training methods for this purpose, the superiority of specific exercise modalities (such as resistance versus endurance exercise programs), or the suitability of these programs for different cancer types [9].

In breast cancer, a recent study by Short et al. [18] evaluated the impact of a 12-week concurrent program (including resistance, endurance, and flexibility training) on PhA in breast cancer survivors (BCS). The study found a statistically significant increase in PhA, which was weakly but directly related to improvements in muscular strength [18]. These results suggest a potential positive effect of exercise in this population, highlighting the need for further studies to expand our understanding and facilitate the integration of these findings into clinical practice.

Additionally, analyzing the correlation between PhA and other BIA parameters and key health outcomes, such as functional tests, could provide insights into how changes in BIA are related to functional improvements, which would be valuable for the introduction of PhA evaluation into clinical settings.

The main aim of this study was to analyze the effects of a 12-week therapeutic exercise intervention on PhA, R, and Xc in BCS. The secondary aim was to investigate the relationship between changes in BIA variables and changes in functional capacity and muscular strength.

## Methods

### Study design and setting

This was a prospective cohort study involving BCS. The data were obtained from a larger clinical trial (NCT03879096) conducted between May 2017 and January 2020 at the University Clinical Hospital Virgen de la Victoria in Málaga (Spain). The study received ethical clearance by the Portal de Ética de la Investigación Biomédica de Andalucía Ethics Committee (2804/2016). The CONSORT checklist was followed to ensure transparent and standardized reporting of the study.

All participants were fully informed about the purpose and content of the investigation and signed a written consent prior to the start of the study. They were informed that they could leave the study at any time and no explanations would be necessary. The study adhered to the principles of the Declaration of Helsinki.

### Participants

Potentially eligible patients were recruited through oncologists from the Medical Oncology Unit of the hospital. Female patients over the age of 18 were offered to participate in the study if they were BCS who had previously undergone surgery for their primary tumor and showed no evidence of recurrence at the time of recruitment. Participants could be under hormonal treatment or undergoing radiotherapy or antiHER therapy. Contrarily, potential participants were excluded if they had a previous history of any cardiovascular event diagnosed as cardiac rhythm disorders, stable or unstable angina, acute pulmonary edema, or syncope of unrelated etiology in the previous year.

### Intervention

A 12-week therapeutic exercise program was implemented, including two weekly sessions, each comprising 30 min of strength exercises and 20 min of endurance training led by a physical therapist. The intervention took place in groups of 7–8 participants. To ensure the program was appropriately individualized, each participant underwent a preliminary functional capacity assessment, allowing for adjustments to the exercise volume, intensity, and complexity according to their specific abilities and needs [25]. In general, for resistance training, the focus during the first 2 weeks was on performing exercises with proper technique. Participants completed 3 sets of 15 repetitions using a load that ensured correct execution. This load was adjusted using dumbbells and/or elastic bands. In subsequent weeks, the volume was increased to 4 sets of 10 repetitions, increasing weights whenever participants could perform more than 12 repetitions with proper technique. In this way, an approximate effort of 10–12 repetition maximum was sought to be guaranteed. For endurance training, the initial 2 weeks were dedicated to acclimating participants to the sensation of fatigue during exercise. Low-intensity exercise (below 60% of the maximum age-predicted heart rate) was employed during this period. In the following weeks, participants were instructed to maintain a consistent pace corresponding to a heart rate range of 60 to 80% of their maximum age-predicted heart rate, which was previously determined through an incremental submaximal test. In case participants experienced any symptoms during exercise, they were instructed to reduce intensity. Every 2 weeks, heart rate and perceived exertion at the selected velocity were assessed to adjust intensity levels in response to any improvements. Further details about the exercise program have been published elsewhere [25].

### Measurements

Measurements were performed at baseline and after 12 weeks. Age, height, weight, and body mass index data were collected as anthropometric information. Additionally, clinically relevant disease-related data were gathered, such as years since diagnosis, the modality of surgical intervention (breast-conserving surgery or mastectomy), and details about previous and current treatments (radiotherapy, chemotherapy, hormone therapy, or monoclonal antibody).

For the purposes of the study, body composition and function-related outcomes were measured using BIA techniques and strength and functional tests, respectively, all of which are described in detail below. All measurements were conducted in the same location and by the same evaluators.

#### Primary outcome: bioelectrical impedance analysis-derived phase angle

Whole-body BIA was assessed using a phase-sensitive single-frequency impedance analyzer (BIA 101 Whole Body Bioimpedance Vector Analyzer (AKERN, Italy)). This device operates as a tetrapolar BIA system, with electrodes placed on the hand and foot, using a frequency of 50 kHz and a measurement current of 800 μA. Body weight and standing height were measured before the BIA procedure using a Tanita TBF- 300 A (TANITA CORPORATION, Tokyo, Japan) and a 2-mm sensitivity laser height rod, respectively. Then, BIA measurements were performed according to the manufacturer’s guidelines [26]. The participant was positioned supine on a hospital bed, with legs positioned 45° from the midline of the body and the upper limbs at a 30° angle from the trunk. After cleansing the skin with isopropyl alcohol, very low-impedance Ag/AgCl electrodes (BIVAtrodes, AKERN, Italy) were placed on the back of the right hand and the right foot (except in the case of unilateral lymphedema on the right side, in which case the electrodes were placed on the opposite hand and foot). Each electrode included a sensor and injector area separated by a distance of 5 cm [27]. To minimize fluid distribution disturbances, participants were instructed not to eat or drink for at least 2 h before the test [28]. Additionally, since fluid shifts occur when moving from a standing to a supine position and can directly affect results, a 5-min waiting period in the supine position was observed before performing the BIA measurements.

Through this procedure, the Xc and R parameters were obtained, and PhA was calculated as the arc-tangent of the ratio Xc/R multiplied by 180°/π.

#### Secondary outcomes: function-related outcomes (functional capacity and hand-grip strength)

Functional capacity was assessed using the 30-STS, in which subjects were instructed to sit and stand from a 43-cm-high chair as quickly as possible, completing the entire motion range while keeping their arms crossed over the chest [29]. The number of repetitions performed in 30 s was recorded as the test result.

The hand grip strength was measured using a Jamar Hydraulic Hand Dynamometer Model SH5001 (Lafayette Instrument, Lafayette, USA), since hand grip dynamometry is considered the gold standard for measuring this parameter [30]. For this purpose, subjects held the dynamometer with their dominant hand (elbow flexed at 90° and wrist in a neutral position) and performed three isometric maximal voluntary contractions, each lasting 5 s, with a 1-min rest between repetitions. During the test, subjects sat on a chair without armrests with their feet on the floor and kept their back straight. The mean of the three trials was recorded as the result of the test (in kg).

### Statistical analysis

Data were tabulated and analyzed using SPSS for Windows (version 25.0, SPSS Inc., Chicago, IL, USA). Quantitative variables were reported as means and standard deviations, while qualitative variables were presented as absolute and relative frequencies (%). In the case of quantitative outcomes, a bivariate analysis was performed to assess mean differences, using the two-tailed paired Student’s *t*-test for dependent samples to compare pre- and post-intervention outcomes. The normality of the data was verified with the Kolmogorov–Smirnov test.

The relationship between changes in PhA and function-related outcomes was assessed using the Pearson’s *r* correlation coefficient. Two separate analyses were carried out, evaluating, on the one hand, the existing correlation between the values obtained at baseline and, on the other hand, the correlation between the values obtained after the 12-week intervention. The results of the post-intervention analysis are presented in the “Results” section of this manuscript, while baseline correlations are available in the Supplementary Appendix 1. The resulting correlations were considered poor (*r* < 0.49), moderate (*r* = 0.50–0.74), or strong (*r* > 0.75) [31].

Additionally, multiple and linear regression analyses were carried out to identify the best model for predicting functional test outcomes (Hand grip and 30-STS tests) based on baseline BIA values (R, Xc, and PhA).

In all analyses, a 95% confidence interval was established, and a *p*-value < 0.05 was considered statistically significant.

## Results

A total of 67 women participated in this prospective study. Treatment of most of the participants included breast-conserving surgery, chemotherapy, and radiotherapy, and most of them were still under hormone therapy. Participants had been diagnosed an average of 2.53 (2.32) years before the onset of the study, with diagnosis ranging from 3 months to 13 years prior. Additional descriptive and clinical data can be found in Table 1.

**Table 1:** Participant descriptive and clinical variables (*n* = 67)

	Mean (SD)	Min–max
**Age (years)**	51.88 (9.51)	32–70
**BMI (kg/m**^**2**^**)**	27.45 (5.39)	17.60–43.50
**Years from diagnosis**	2.53 (2.32)	0–13
**Surgical intervention**		**Percentage (*n*)**
	Breast-conserving surgery	64.2% (43)
	Mastectomy	34.3% (23)
**Cancer treatment**		
	Chemotherapy	86.6% (58)
	Radiotherapy	78.4% (52)
	Hormone therapy	77.6% (50)
	Monoclonal antibody	32.8% (22)
**Current treatment**		
	None	23.9% (16)
	Hormone therapy	59.7% (40)
	Hormone therapy and monoclonal antibody	4.5% (3)
	Chemotherapy	3.0% (2)
	Hormone therapy and monoclonal antibody	4.5% (3)
	Monoclonal antibody	3.0% (2)
	Radiotherapy	1.5% (1)

Table 2 presents the R, Xc, and PhA values obtained through the BIA, along with the strength and functional capacity values derived from the Hand grip and 30-STS tests. Regarding BIA results, PhA and R improved significantly after 12 weeks of intervention. In the case of functional and strength tests, only the 30-STS test showed a statistically significant improvement.

**Table 2:** Baseline and post-intervention values of bioimpedance analysis, hand grip strength, and functional capacity, including mean differences and results of the Student’s *t*-test

Outcomes	Baseline	12 weeks (post)	Difference (12 weeks – baseline)	*t*	*p*
	Mean (SD)	Mean (SD)			
**Bioelectrical impedance analysis**					
**Resistance (*R*)**	576.13 (69.95)	563.82 (68.14)	− 12.31 (31.75)	− 3.17**	0.002
**Reactance (Xc)**	51.35 (10.76)	52.19 (8.35)	0.83 (7.71)	0.89	0.378
**Phase angle**	5.08 (0.80)	5.29 (0.62)	0.21 (0.67)	2.64*	0.010
**Functional assessments**					
**30-STS (*n*)**	18.9 (6.0)	25.9 (5.5)	7.1 (5.0)	10.56**	0.000
**Hand grip (kg)**	21.6 (5.5)	22.5 (4.9)	0.9 (3.8)	1.83	0.072

^*^p < 0.05; **p < 0.01; *30-STS* 30 s sit-to-stand test

### Correlations

Regarding correlation analysis, bivariate correlations revealed that PhA values showed the strongest associations with functional outcomes. Specifically, PhA exhibited a significant positive correlation with both hand grip strength and 30-STS tests (*r* = 0.37 [*p* = 0.002] and *r* = 0.39 [*p* = 0.002], respectively). Among the raw BIA parameters, Xc also showed a significant positive correlation with both hand grip strength and 30-STS tests (*r* = 0.28 [*p* = 0.022] and *r* = 0.36 [*p* = 0.004], respectively). In contrast, R did not correlate with any of the strength or functional capacity tests. Expanded data is available in Table 3.

**Table 3:** Pearson correlations (*r*) between bioelectrical impedance analysis (resistance, reactance, phase angle) and strength (hand grip test) and functional (30-sit-to-stand test) outcomes after intervention

	Hand grip test		30-STS test	
	Pearson’s correlation (***r***)	***p***	Pearson’s correlation (***r***)	***p***
**BIA outcomes**				
**Resistance, *R* (Ohm)**	0.00	0.995	0.10	0.440
**Reactance, Xc (Ohm)**	0.28*	0.022	0.36**	0.004
**Phase angle (°)**	0.37**	0.002	0.39**	0.002

^*^p < 0.05; **p < 0.01; *BIA* bioelectrical impedance analysis; *30-STS* 30 sit-to-stand test

### Regression models

The multiple regression model for the hand grip strength test (post-intervention values) was found to be statistically significant (*F* = 9.174; *p* < 0.001). It revealed that baseline PhA values adjusted by age explained 21.4% of the variance in post-intervention hand grip strength test scores (*r* = 0.490, *R*^2^ = 0.240, adjusted *R*^2^ = 0.214). Similarly, baseline Xc values adjusted by age explained 11.3% of the variance in post-intervention hand grip strength test. However, the regression model for baseline *R* values was not found to be statistically significant. Similarly, the multiple regression model developed for the 30-STS test (post-intervention values) also showed statistical significance (*F* = 11.896; *p* < 0.001). This model indicated that baseline PhA values adjusted by age accounted for 28.8% of the variance in post-intervention 30-STS test results (*r* = 0.560, *R*^2^ = 0.314, adjusted *R*^2^ = 0.288). Similarly, baseline R and Xc values adjusted by age explained 14.1% and 26.1% of the variance in post-intervention 30-STS test, respectively. Expanded data is available in Table 4. Results from linear regression models between functional and BIA variables, as well as multiple regression models adjusted by body mass index, are shown in Supplementary Appendix 1.

**Table 4:** Results of multiple regression analyses between functional variables and age-adjusted bioimpedance analysis variables

Dependent variables	Predictor variables	Standardized β	*R*	*R*^2^	Adjusted *R*^2^	*p*
**Hand grip strength test** **(12 weeks)**			0.490	**0.240**	**0.214**	< 0.001
	Phase angle (baseline)	0.415				< 0.001
	Age	− 0.234				0.046
**Hand grip strength test** **(12 weeks)**			0.278	0.077	0.046	0.097
	Resistance (baseline)	− 0.094				0.478
	Age	− 0.290				0.031
**Hand grip strength test** **(12 weeks)**			0.378	**0.143**	0.113*	0.012
	Reactance (baseline)	0.277				0.030
	Age	− 0.205				0.105
**30 sit-to-stand test** **(12 weeks)**			0.560	**0.314**	**0.288**	< 0.001
	Phase angle (baseline)	0.388				0.001
	Age	− 0.381				0.002
**30 Sit-To-Stand test** **(12 weeks)**			0.416	**0.173**	0.141**	0.007
	Resistance (baseline)	0.098				0.453
	Age	− 0.384				0.004
**30 sit-to-stand test** **(12 weeks)**			0.537	**0.289**	0.261**	< 0.001
	Reactance (baseline)	0.358				0.004
	Age	− 0.345				0.005

^*^p < 0.05, **p < 0.01

## Discussion

The aims of this study were to analyze the effects of a 12-week therapeutic exercise intervention on PhA, R, and Xc in BCS and investigate the relationship between changes in BIA variables and changes in functional capacity and muscular strength. The main finding was the presence of statistically significant differences in PhA and R measurements before and after the exercise intervention. Additionally, statistically significant correlations were found both for PhA and Xc values with the 30-STS and hand grip strength tests. Finally, baseline PhA, R, and Xc values, all age-adjusted, explained part of the 30-STS variance at 12 weeks, while baseline PhA and Xc values, adjusted by age, explained part of the hand grip strength test variance post-intervention.

Concerning the changes observed after the intervention, the statistically significant increase in PhA aligns with current evidence, which suggests that this parameter increases following this type of approach. Thus, previous studies also reported similar PhA improvements with the implementation of a concurrent exercise program in this population [18, 32]. This increase in PhA is similar to those found in a study that applied a nutritional intervention in breast cancer patients [13]. However, and in contrast, two studies reported no changes in PhA after a 10-week yoga intervention [33] and an unsupervised 6-month resistance and aerobic training program [34], respectively, despite improved fitness in the second study. These data may indicate the need to reach appropriate thresholds in intensity, volume, duration, and/or adherence to the program to improve BIA parameters, as has been previously suggested in a previous review in patients with cancer [9]. This hypothesis may be supported by the lack of significant differences in PhA in studies that used lower intensities in other cancer survivors populations [9]. Thus, as suggested by some authors, the application of moderate to high training intensities may be a key factor to obtaining positive results [18]. In this line, it has previously been proposed that higher volumes and intensities are drivers of hypertrophy and metabolic stress, which would ultimately improve membrane integrity, leading to enhancements in Xc and PhA [18, 35].

Regarding the exercise modality associated with better outcomes, the evidence remains unclear. However, a potential superiority of resistance training over endurance training has been suggested. This hypothesis is also supported by the findings of Short et al. [18], who observed changes in resistance parameters but not in cardiorespiratory ones applying a combined program in breast cancer survivors that resulted in increased PhA [18]. In addition, evidence in other populations (e.g., athletes) has also shown a greater increase in PhA with resistance training compared to endurance training [36]. Considering the above, concurrent resistance and endurance training programs with high intensity appear feasible and beneficial for this population. However, future studies should delineate the contribution of each training modality to the observed effects.

Interestingly, in contrast to existing literature, our study found a statistically significant decrease in R, but no significant changes in Xc. It has been previously suggested that exercise might increase Xc through adaptations that improve cell membrane integrity, while nutritional interventions may have a greater impact on R by increasing of electrolyte-containing water [24]. The results of our study could suggest a systemic effect of exercise that indirectly affects R or the participation of other factors in patients’ recovery that influence the observed changes, including a possible change in diet and/or lifestyle. However, although a trend toward improvement in Xc was observed in our study, adjusting some intervention parameters could be necessary to achieve significant results in this specific parameter. In the case of the study by Short et al. [18], no statistically significant changes were found in any of the two raw parameters (R and Xc) despite the increase in PhA. The data from this study, showing improvements in PhA values accompanied by functional gains without significant differences in one or both raw parameters, may suggest that focusing on PhA rather than raw parameters could be more informative. Future research should therefore confirm whether our findings regarding raw parameters are coincidental or have a meaningful explanation in this specific population.

Interestingly, and in line with the above, the changes observed in our study using BIA were consistent with functional improvements, as evidenced by a statistically significant increase in post-intervention values for the 30-STS test. This relationship must be interpreted in the context of the results of previous studies like Mascherini et al. [34], who, as described above, obtained an improvement in the hand grip strength, 30-STS after a 6-month exercise program, without achieving statistically significant changes in the PhA. In any case, in the sphere of patients’ well-being and regardless of the BIA data, the recovery of functional capacity and muscular strength, which are often diminished during disease and treatment, is particularly relevant as it directly impacts prognosis and quality of life [14, 37, 38]. In this regard, exercise programs clearly seem to play a crucial role in this population in the recovery of these outcomes, among other key health indicators [37, 39].

In our study, the positive correlations of PhA and Xc found with hand grip strength and 30-STS tests provide valuable insights into the impact of body composition changes, as measured by BIA, on functional capacity. Previous studies, such as that by Short et al. (2022), have also found correlations between PhA and functional tests. However, the tests used differ from those used in our study, making direct comparison difficult. Nonetheless, these relationships are consistent with current literature, which proposes that PhA can be considered a marker of muscle strength and functionality in BCS [14] and other cancer patients [40]. It is worth nothing that these correlations were not found to the same extent in the analysis of the baseline values of our study, where only was found a correlation between PhA and hand grip strength test (data available in Supplementary Appendix 2). While these data must be confirmed by other studies, it could be hypothesized that patient’s recovery, aided by exercise, could help normalize the state of body tissues, thereby improving their association with the patient’s functional capacity.

Finally, the multiple regression analyses revealed two statistically significant models, with baseline PhA values adjusted by age explaining 21.4% and 28.8% of the variance in the hand grip strength and 30-STS tests, respectively. These models were similarly replicated for the baseline Xc values adjusted by age (explaining 11.3% and 26.1% of the variances, respectively), while they could only be replicated in the case of the baseline R values with the 30-STS test (14.1%). These results align with other studies that have suggested PhA as a predictor of maximal forearm isometric strength and a potential indicator of disease-related functionality in breast cancer patients [14]. These data are of particular interest from a prognostic and clinical prediction perspective, as they indicate that baseline PhA, as well as the raw parameters Xc and R, could reflect functional capacity response to intervention.

### Strengths and limitations of the study

Some strengths and limitations of this study should be considered. First, as a limitation, the single-group design prevents the establishment of a cause-and-effect relationship. Moreover, the dietary and physical activity behaviors beyond the exercise sessions were not recorded, limiting our ability to fully attribute the observed outcomes to the intervention alone. Although the inclusion of participants with lymphedema increases the generalizability of the results, as this is a frequent sign in this population, it can also be a limitation due to its influence on the results of BIA measurements. Finally, our results cannot be generalized to populations other than BCS. A key strength, however, is the inclusion of both raw parameters (R and Xc) alongside PhA, which provides a more comprehensive analysis of the mechanisms of action while eliminating potential BIA-related bias from using predictive equations based on reference models.

### Future research

In light of the results obtained and current literature, further research is warranted to confirm the observed effects of exercise on PhA and specific raw parameters such as R and Xc through controlled studies and to elucidate the underlying mechanisms. Future studies should also investigate the importance of different training modalities in the resulting benefits.

## Conclusions

The main finding of this study is that a 12-week exercise program including resistance and endurance training may effectively improve PhA and R. Additionally, statistically significant correlations were observed both for PhA and Xc values with functional and strength capacity outcomes (30-STS and hand grip strength tests). Finally, baseline PhA, Xc, and R values explained part of the 30-STS and hand grip strength tests variance at 12 weeks, which could suggest its potential prognostic value for these patients.

## Supplementary Information

Below is the link to the electronic supplementary material.

Supplementary file1 (DOCX 25 KB)

## Data Availability

The data are available from the authors upon reasonable request.

## Abbreviations

BIA: Bioelectrical impedance analysis
BCS: Breast cancer survivors
PhA: Bioelectrical impedance analysis-derived phase angle
R: Resistance
Xc: Reactance
30-STS: 30-Second sit-to-stand test
