# Supplementary material for: Effect of a 12-week exercise program on phase angle in women with breast cancer
Source: Support Care Cancer. 2025 Apr 21;33(5):398. doi: 10.1007/s00520-025-09443-4 (PMC12011936; doi:10.1007/s00520-025-09443-4)
Supplement: Supplementary file 1 — Supplementary file1 (DOCX 25 KB) [file 520_2025_9443_MOESM1_ESM.docx]

**SUPPLEMENTARY APPENDIX**

**SUPPORTIVE CARE IN CANCER**

Supplement to: ***Effect of a 12-week exercise program on phase angle in women with breast cancer***

Adrian Escriche-Escuder^1,2^, José Manuel García-Almeida^2,3^, Isabel María Vegas-Aguilar^2,3^, Bella Pajares^2,4^, Emilio Alba^2,4^, Manuel Trinidad-Fernández^1,2^, Cristina Roldán-Jiménez^1,2^, Antonio Ignacio Cuesta-Vargas^1,2*^

**Affiliations**

^1^Department of Physiotherapy, University of Malaga, Málaga, Spain

^2^Instituto de Investigación Biomédica de Málaga (IBIMA), Málaga, Spain

^3^Department of Endocrinology and Nutrition, Hospital Regional Universitario y Virgen de la Victoria, Málaga, Spain

^4^UGCI Oncológica Médica. Hospital Regional Universitario y Virgen de la Victoria, Málaga, Spain

**Corresponding author:** Antonio I. Cuesta-Vargas; [acuesta@uma.es](mailto:acuesta@uma.es); Departamento de Fisioterapia, Universidad de Málaga. C/ Arquitecto Peñalosa, 3. PC: 29071. Malaga (Spain)

**Supplementary Appendix 1.** Multiple and linear regression analysis not included in the main document.

**Table S.1.** Linear regression analysis about 30-Sit-To-Stand test and bioelectrical impedance analysis variables.

| Dependent variables | Predictor variables | Standardized β | r | R^2^ | F | p |
| --- | --- | --- | --- | --- | --- | --- |
| 30 Sit-To-Stand test  (12 weeks) | Phase Angle (Baseline) | 0.341 | 0.341 | **0.116**** | 7.772 | 0.007 |
| 30 Sit-To-Stand test  (12 weeks) | Resistance (Baseline) | 0.138 | 0.138 | 0.019 | 1.138 | 0.290 |
| 30 Sit-To-Stand test  (12 weeks) | Reactance (Baseline) | 0.335 | 0.335 | **0.112**** | 7.446 | 0.008 |

**p<0.01

**Table S.2.** Linear regression analysis about Hand grip strength test and bioelectrical impedance analysis variables.

| Dependent variables | Predictor variables | Standardized β | r | R^2^ | F | p |
| --- | --- | --- | --- | --- | --- | --- |
| Hand grip strength test  (12 weeks) | Phase Angle (Baseline) | 0.356 | 0.356 | **0.126*** | 9.413 | 0.003 |
| Hand grip strength test  (12 weeks) | Resistance (Baseline) | -0.46 | 0.046 | 0.002 | 0.135 | 0.714 |
| Hand grip strength test  (12 weeks) | Reactance (Baseline) | 0.244 | 0.244 | **0.060*** | 4.123 | 0.046 |

*p<0.05, **p<0.01

**Table S.3.** Results of multiple regression analyses between functional variables and BMI-adjusted bioimpedance analysis variables.

| Dependent variables | Predictor variables | Standardized β | r | R^2^ | Adjusted R^2^ | p |
| --- | --- | --- | --- | --- | --- | --- |
| Hand grip strength test  (12 weeks) |  |  | 0.359 | **0.129** | **0.101*** | 0.012 |
|  | Phase Angle (Baseline) | 0.349 |  |  |  | 0.004 |
|  | BMI | -0.046 |  |  |  | 0.699 |
| Hand grip strength test  (12 weeks) |  |  | 0.198 | 0.039 | 0.009 | 0.278 |
|  | Resistance (Baseline) | -0.250 |  |  |  | 0.167 |
|  | BMI | -0.281 |  |  |  | 0.121 |
| Hand grip strength test  (12 weeks) |  |  | 0.246 | 0.061 | 0.031 | 0.136 |
|  | Reactance (Baseline) | 0.262 |  |  |  | 0.068 |
|  | BMI | 0.034 |  |  |  | 0.810 |
| 30 Sit-To-Stand test  (12 weeks) |  |  | 0.500 | **0.250** | **0.224**** | <0.001 |
|  | Phase Angle (Baseline) | 0.286 |  |  |  | 0.016 |
|  | BMI | -0.370 |  |  |  | 0.002 |
| 30 Sit-To-Stand test  (12 weeks) |  |  | 0.460 | **0.211** | **0.184**** | 0.001 |
|  | Resistance (Baseline) | -0.282 |  |  |  | 0.086 |
|  | BMI | -0.607 |  |  |  | <0.001 |
| 30 Sit-To-Stand test  (12 weeks) |  |  | 0.442 | **0.195** | **0.167**** | 0.002 |
|  | Reactance (Baseline) | 0.180 |  |  |  | 0.184 |
|  | BMI | -0.327 |  |  |  | 0.018 |

*p<0.05, **p<0.01

**Supplementary Appendix 2.** Pearson correlations (r) between Bioelectrical Impedance Analysis (Resistance, Reactance, Phase Angle) and strength (Hand Grip test) and functional (30-Sit-To-Stand test) outcomes before intervention (baseline values).

|  | Hand grip strength test | | 30-STS test | |
| --- | --- | --- | --- | --- |
|  | **Pearson’s correlation (r)** | **p** | **Pearson’s correlation (r)** | **p** |
| BIA outcomes |  | | | |
| Resistance (Ohm) | -0.71 | 0.569 | 0.01 | 0.940 |
| Reactance (Ohm) | 0.18 | 0.159 | 0.16 | 0.206 |
| Phase Angle (°) | 0.28* | 0.023 | 0.20 | 0.115 |
